# Biallelic *NDC1* variants that interfere with ALADIN binding are associated with neuropathy and triple A-like syndrome

**DOI:** 10.1016/j.xhgg.2024.100327

**Published:** 2024-07-14

**Authors:** Daphne J. Smits, Jordy Dekker, Hannie Douben, Rachel Schot, Helen Magee, Somayeh Bakhtiari, Katrin Koehler, Angela Huebner, Markus Schuelke, Hossein Darvish, Shohreh Vosoogh, Abbas Tafakhori, Melika Jameie, Ehsan Taghiabadi, Yana Wilson, Margit Shah, Marjon A. van Slegtenhorst, Evita G. Medici-van den Herik, Tjakko J. van Ham, Michael C. Kruer, Grazia M.S. Mancini

**Affiliations:** 1 Department of Clinical Genetics, Erasmus University Medical Center, Dr. Molewaterplein 40, 3015 GD Rotterdam, the Netherlands; 2 Division of Pediatric Neurology, Barrow Neurological Institute, Phoenix Children’s Hospital, Phoenix, AZ 85016, USA; 3 Departments of Child Health, Neurology, Cellular & Molecular Medicine and Program in Genetics, University of Arizona College of Medicine - Phoenix, Phoenix, AZ 85004, USA; 4 Department of Pediatrics, Faculty of Medicine and University Hospital Carl Gustav Carus, Technische Universität Dresden, Dresden, Germany; 5 Department of Neuropediatrics and NeuroCure Clinical Research Center, Charité-Universitätsmedizin Berlin, Berlin, Germany; 6 Neuroscience Research Center, Faculty of Medicine, Golestan University of Medical Sciences, Gorgan, Iran; 7 Clinical Research Development Unit (CRDU), Sayad Shirazi Hospital, Golestan University of Medical Sciences, Gorgan, Iran; 8 Iranian Center of Neurological Research, Neuroscience Institute, Tehran University of Medical Sciences, Tehran, Iran; 9 Skin and Stem Cell Research Center, Tehran University of Medical Sciences, Tehran, Iran; 10 Sydney Medical School, Faculty of Medicine and Health, The University of Sydney, Sydney, NSW, Australia; 11 Cerebral Palsy Alliance Research Institute, Faculty of Medicine and Health, The University of Sydney, Sydney, NSW, Australia; 12 Department of Clinical Genetics, Children’s Hospital at Westmead, Sydney Children’s Hospitals Network, Westmead, NSW, Australia; 13 Specialty of Genomic Medicine, Faculty of Medicine and Health, University of Sydney, Sydney, NSW, Australia; 14 Department of Neurology, Section of Child Neurology, Erasmus University Medical Center Rotterdam, Dr. Molewaterplein 40, 3015 GD Rotterdam, the Netherlands

**Keywords:** NDC1, ALADIN, triple A syndrome, nuclear pore complex, peripheral neuropathy, alacrima, achalasia, neurodevelopmental disorder

## Abstract

Nuclear pore complexes (NPCs) regulate nucleocytoplasmic transport and are anchored in the nuclear envelope by the transmembrane nucleoporin NDC1. NDC1 is essential for post-mitotic NPC assembly and the recruitment of ALADIN to the nuclear envelope. While no human disorder has been associated to one of the three transmembrane nucleoporins, biallelic variants in *AAAS*, encoding ALADIN, cause triple A syndrome (Allgrove syndrome). Triple A syndrome, characterized by alacrima, achalasia, and adrenal insufficiency, often includes progressive demyelinating polyneuropathy and other neurological complaints. In this report, diagnostic exome and/or RNA sequencing was performed in seven individuals from four unrelated consanguineous families with *AAAS*-negative triple A syndrome. Molecular and clinical studies followed to elucidate the pathogenic mechanism. The affected individuals presented with intellectual disability, motor impairment, severe demyelinating with secondary axonal polyneuropathy, alacrima, and achalasia. None of the affected individuals has adrenal insufficiency. All individuals presented with biallelic *NDC1* in-frame deletions or missense variants that affect amino acids and protein domains required for ALADIN binding. No other significant variants associated with the phenotypic features were reported. Skin fibroblasts derived from affected individuals show decreased recruitment of ALADIN to the NE and decreased post-mitotic NPC insertion, confirming pathogenicity of the variants. Taken together, our results implicate biallelic *NDC1* variants in the pathogenesis of polyneuropathy and a triple A-like disorder without adrenal insufficiency, by interfering with physiological NDC1 functions, including the recruitment of ALADIN to the NPC.

## Teaser

This report describes the identification of biallelic *NDC1* variants in children with a triple A-like syndrome, including intellectual disability, motor impairment, severe demyelinating with secondary axonal polyneuropathy, alacrima, and achalasia. The identified *NDC1* variants interfere with physiological NDC1 functions, including the recruitment of ALADIN to the nuclear pore complex.

## Introduction

The nuclear envelope (NE) is a specialized membrane that separates the nucleus from the cytoplasm. At sites where the inner and outer nuclear membrane fuse, nuclear pore complexes (NPCs) are embedded in the NE. The NPC consist of several multiprotein subcomplexes that are anchored in the membrane via three transmembrane nucleoporins: NDC1, POM121, and GP210.^1^ Altogether, the NPC subcomplexes form a cylindrical ring structure, which is essential for their most important functions: facilitating nucleocytoplasmic transport of proteins, RNAs, and other signaling molecules.^2^ In addition, transmembrane nucleoporins are involved in multiple other cellular processes such as transcriptional regulation, chromatin organization, and mitotic progression.^3^

Given their diverse functions, variants in genes encoding nucleoporins have been associated with a wide variety of disorders, including infantile bilateral striatal necrosis, nephrotic syndrome, and neurodevelopmental disorders.^4^^,^^5^^,^^6^ No disease-related variants have been described for the three essential transmembrane nucleoporins *NDC1*, *POM121*, and *GP210*. However, their upregulation is associated with various cancer types, including non-small cell lung cancer, cervical cancer, and esophageal squamous cell carcinoma.^7^^,^^8^^,^^9^ Increased NDC1 expression and mislocalization is also described in ventricular cardiac tissue of ischemic cardiomyopathy and dilated cardiomyopathy patients.^10^ In mice, a variant in *NDC1* resulted in gametogenesis defects and skeletal malformations.^11^

NDC1 is a transmembrane nucleoporin that is essential for post-mitotic NE formation and NPC assembly.^12^^,^^13^ During the first steps of NE/NPC assembly, NDC1 is anchored in the NE via six transmembrane domains. Both its N and C termini face the NPC channel and facilitate the recruitment of other nucleoporins and membrane remodeling factors. The highly conserved large C-terminal tail of NDC1 recruits the nucleoporin ALADIN. At the NPC, ALADIN regulates selective nuclear import and is therefore essential for cellular homeostasis. Variants in *AAAS*, encoding for ALADIN, cause triple A syndrome, also known as Allgrove syndrome (OMIM: 231550). Triple A syndrome is an autosomal recessive disorder characterized by ACTH-resistant adrenal insufficiency, alacrima, achalasia, and a variety of neurological disturbances.^14^^,^^15^^,^^16^^,^^17^^,^^18^ In individuals with triple A syndrome, mislocalization of ALADIN is observed and is thought to be the main pathogenic mechanism underlying the disease.^19^^,^^20^^,^^21^

Here, we describe the first cohort of seven individuals, from four unrelated families, with biallelic variants in *NDC1*. The affected individuals present with alacrima, achalasia, and neurological features including developmental delay/intellectual disability, demyelinating and secondary axonal polyneuropathy, facial weakness, tongue fasciculation, and hypotonia, resembling the features observed in triple A syndrome. Cell biological studies were performed to provide insights into the molecular mechanisms contributing to disease pathogenesis.

## Material and methods

### Standard protocol approvals, registrations, and patient consents

The study was approved by the local ethics boards/IRBs. Clinical data from affected individuals were recruited through GeneMatcher.^22^ Written informed consent to participate in this study was obtained from all affected individuals or their parents/caretakers. Written informed consent for publication of photographs was obtained as applicable. Fibroblasts from affected individuals were obtained from skin biopsies previously sampled for routine diagnostics.

### Exome sequencing

Exome sequencing was performed on DNA isolated from blood derived from probands and family members in different laboratories. Whole exome sequencing (WES) data are deposited internally in each medical institute, in respect to the privacy of the families. Details of sequencing and analysis pipelines are described in the supplemental methods.

### RNA sequencing

The RNA sequencing pipeline used for family 1, individuals 1-I and 1-II, is described in Dekker et al.^23^ In short, skin biopsy-derived fibroblasts were cultured in Ham’s F10 medium containing 15% (v/v) fetal calf serum and 1% (v/v) penicillin/streptomycin. Fibroblast cultures for RNA sequencing were either untreated or treated with 100 μg/mL cycloheximide (Sigma-Aldrich) for 24 h. At a confluency of 70%–80%, RNA was isolated and RNA samples were enriched for polyadenylated RNA, followed by cDNA synthesis. Strand-specific sequencing was performed on an Illumina NovaSeq 6000 with 150 bp paired-end reads and unique molecular identifier-adaptors, generating a minimum of 40 million reads. Mapped RNA sequencing reads were visualized in the Integrative Genomics Viewer (IGV, v.2.14.5) using hg19 as reference genome.

### Minigene assay

To confirm effects on splicing, a minigene exon-trapping assay was performed. *NDC1* exon 9 and surrounding intronic sequences (∼100 bp upstream and downstream of splice donor and acceptor site) were amplified from genomic DNA and cloned into the pSPL3 vector (Thermo Fisher Scientific, Invitrogen) using Gibson assembly. HEK293T cells (70%–90% confluent) growing in a 12-well plate were transfected with 1 μg of the minigene constructed using 5 μg polyethyleneimine. After 24 and 48 h incubation, RNA was isolated using an RNeasy mini kit (QIAGEN, Venlo, the Netherlands), according to the manufacturer’s protocol, followed by cDNA synthesis using the iScript cDNA synthesis kit (Bio-Rad). Transcribed products from the vector were amplified using a standard set of primers. These RT-PCR products were visualized by agarose gel electrophoresis and confirmed by Sanger sequencing.

### Immunofluorescence

Fibroblast cells were grown in Dulbecco’s modified Eagle’s medium supplemented with 10% (v/v) fetal calf serum and 1% (v/v) penicillin/streptomycin. Cells were regularly checked for mycoplasma infections. For immunostainings, fibroblast cells were grown on 24 mm coverslips (Thermo Fisher Scientific). Cells were fixed in 4% paraformaldehyde for 20 min on ice. After fixation, coverslips were blocked for 1.5 h on ice in blocking buffer containing 50 mM Tris-HCl (pH 7.4), 0.9% NaCl, 0.25% gelatin, 0.5% Triton X-100. Primary antibodies were diluted in blocking buffer and incubated overnight at 4°C. The next day, cells were incubated for 45 min with secondary antibodies and mounted on microscopy slides with ProLong Gold Antifade Reagent with DAPI (Thermo Fisher Scientific).

### Antibodies

Primary antibodies: mouse anti-ALADIN (1:100, Santa Cruz, sc-374073), mouse anti-414 (MMS-120P, 1:500, BioLegend), and rabbit anti-CDT1 (D10F11, 1:200, Cell Signaling Technologies).

Secondary antibodies: goat anti-rabbit IgG (H + L) Alexa Fluor 488 (1:200, Thermo Fisher Scientific, A11088), Cy3 AffiniPure donkey anti-mouse IgG (H + L) (1:100, Jackson Laboratory, 715-165-150).

### Confocal microscopy

Confocal fluorescent z stack images were acquired with the Broadband Leica TCS SP5, using Leica LAS AF application (Leica Microsystems). Lasers with 405, 488, and 561 nm excitation wavelength were used to visualize fluorescent secondary antibodies. Maximal projections of z stack images were processed with Fiji ImageJ software.

Super-resolution 3D-SIM microscopy was performed with the Elyra Zeiss PS1. Images were acquired using a 63× 1.4 NA oil objective, 405, 488, and 561 nm diode lasers with appropriate filters. The grating present in the light path was modulated in five phases and five rotations, and multiple z slices with an interval of 110 nm were recorded on an Andor iXon DU 885, 1002 × 1004 EMCCD camera. Raw images were reconstructed using the Zeiss Zen software and subsequently analyzed with Fiji ImageJ software.

## Results

**Figure 1:**
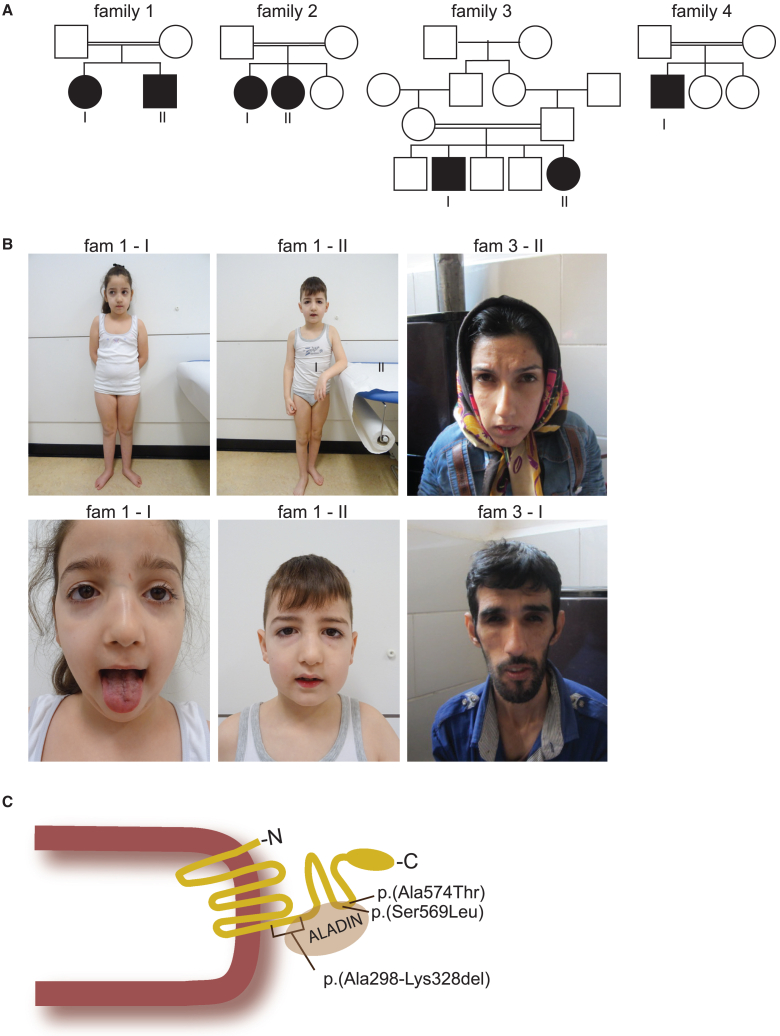
Clinical features of affected individuals with *NDC1* variants (A) Pedigrees of four families with biallelic *NDC1* variants. Affected individuals are depicted in black. (B) Facial features of affected individuals 1-I, 1-II, 3-I, and 3-II. The lower panel in individual 1-I shows tongue fasciculations. (C) Schematic drawing of the NDC1 protein in the nuclear envelope. The identified *NDC1* variants and the interaction sites with ALADIN are depicted in the C-terminal NDC1 protein structure.

Genomic analysis, through WES or RNA sequencing, detected homozygous *NDC1* variants in a cohort of seven individuals from four unrelated families (Figure 1A). Phenotypic features were very similar and are summarized in Table 1, while detailed clinical reports and additional information on the genomic alterations are provided in the supplemental information and Tables S1 and S2.

**Table 1:** Summary of clinical features observed in individuals with biallelic *NDC1* variants

	Ind 1-I	Ind 1-II	Ind 2-I	Ind 2-II	Ind 3-I	Ind 3-II	Ind 4	% of individuals
Variant in *NDC1*: NM_018087.5	c.892-21G>A p.Ala298_Lys328del	c.892-21G>A p.Ala298_Lys328del	c.892-21G>A p.Ala298_Lys328del	c.892-21G>A p.Ala298_Lys328del	c.1706C>T p.Ser569Leu	c.1706C>T p.Ser569Leu	c.1720G>A p.Ala574Thr	
Age at last investigation (years)	12	10	13	10	±25	26	12	
ID	borderline	borderline	mild	no	moderate	moderate	severe	100
Motor impairment	motor delay	motor delay	motor impairment	motor impairment	motor impairment	motor impairment	severe motor impairment	100
Clinical signs of polyneuropathy/EMG	yes (EMG confirmation)	yes (EMG confirmation)	yes (EMG confirmation)	yes, no EMG	yes, no EMG	yes, no EMG	no	86
Alacrima	yes	yes	yes	yes	not reported	yes	no	71
Dysphagia/achalasia	dysphagia, recurrent choking	suspect achalasia	no	no	achalasia	achalasia	dysphagia	71
Facial weakness	yes	yes	yes	not reported	yes	yes	not reported	71
Hypotonia	yes	yes	not reported	not reported	not reported	yes	yes	57
Tongue fasciculations	yes	yes	yes	yes	no	no	no	57
Growth delay	no	no	yes	yes	yes	yes	no	57
Epilepsy	no	no	no	no	no	yes	yes	28
Other autonomic features	no	no	yes	yes	no	no	no	28

EMG, electromyography; ID, intellectual disability.

### Clinical description

All described individuals were born after uncomplicated pregnancies with growth measurements within the normal range. In all, symptoms started in the first year of life, characterized by delayed motor development in most individuals. The individuals from family 3 initially presented with vomiting and recurrent aspiration, but also experienced delayed motor development in early childhood. All individuals had intellectual disability, with variable severity. Speech development was affected and ranged between normal and non-verbal. Nasal speech was frequently described (4/7), and often associated with facial weakness (Figure 1B). Feeding difficulties were described for 5/7 affected individuals, and were characterized by dysphagia and near chocking. In one individual achalasia was radiologically confirmed and treated with surgery.

Physical examination showed the involvement of the peripheral, central, and autonomic nervous system. Clinical signs of peripheral neuropathy were observed in all but one individual, and were confirmed with EMG in three individuals. EMG recordings showed a motor demyelinating polyneuropathy with secondary axonal injury. The most frequently reported peripheral neurological features were hypotonia, muscle atrophy, decreased muscle strength, and decreased deep tendon reflexes. Other lower motor neuron signs included facial weakness (5/7), tongue atrophy, and tongue fasciculations (4/7). Exceptionally, individual 4-I presented with increased muscle tone, contractures of the knees, elbows, and ankles. He also developed epilepsy in the first year of life, characterized by atonic/tonic episodes or head drops. Epilepsy was also reported for individual 3-II. Dysfunction of the autonomic nervous system was seen in 5/7 individuals. Alacrima or hypolacrima was the most frequent autonomic feature and present in 5/7 individuals. In addition, limited heart rate variability was observed in two individuals.

The most frequently described facial features are related to facial muscle weakness, with downslant of the eyelids, a long thin nose, a high arched palate and a pre-auricular tag (Figure 1B). Examination of the hands and feet showed tapered fingers (3/7), hypotrophy of the muscles on the hands and feet (4/7), and hyperkeratosis (2/7). Remarkably, none of the affected individuals had adrenal insufficiency. Baseline cortisol levels were normal and normal responses to ACTH stimulation were observed. Brain MRI showed no major structural abnormalities except mild dilatation of the ventricles and prominent perivascular spaces in two individuals. Muscle CT in individual 1-I showed normal musculature of neck and shoulder, mild atrophy of the pelvis muscles, moderate atrophy of the hamstrings, and severe atrophy with fatty infiltration of the gastrocnemius and soleus muscles.

### Genomic analysis

**Figure 2:**
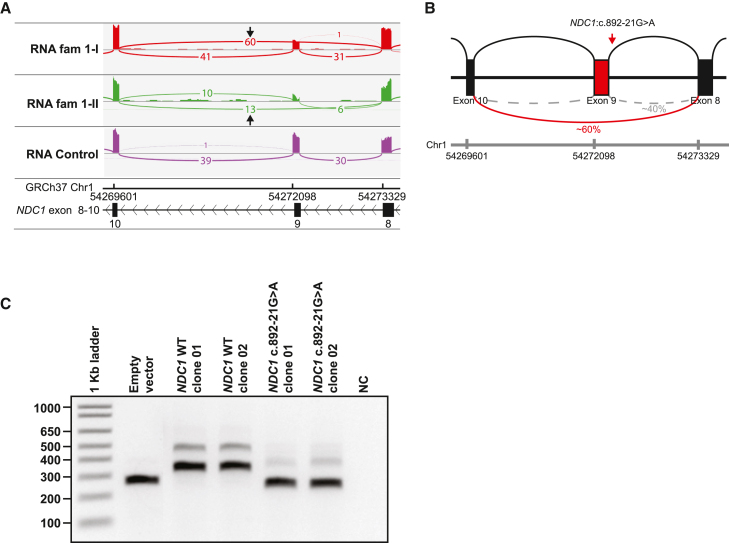
Functional analysis of the variant identified in family 1 (A) Sashimi plot showing partial skipping of exon 9 *NDC1* in both affected individuals from family 1. These samples were not treated with cycloheximide (CHX–). (B) Schematic representation of the effect of the homozygous *NDC1*:c.892-21G>A variant on splicing. In approximately 60% of the reads skipping of exon 9 is observed. (C) Minigene assay confirming the effect of the *NDC1*:c.892-21G>A variant on splicing. HEK293T cells were transfected with plasmid DNA isolated from two independent clones containing *NDC1* with the c.892-21G>A variant, two clones with WT NDC1 sequence, or an empty vector. RT-PCR of the transcribed minigene was performed showing that the *NDC1*:c.892-21G>A induces skipping of exon 9 not seen in WT samples. NC, negative control.

In family 1, diagnostic WES and SNP arrays did not identify pathogenic variants in both siblings, which could explain the phenotype (Table S3). SNP array showed multiple regions of homozygosity and a maternally inherited 215 kb duplication on chromosome 3q13, most likely unrelated to the phenotype. Diagnostic RNA sequencing analysis on both siblings of the overlapping ROH revealed reduced expression of exon 9 of NM_018087.4 (*NDC1*) (Figures 2A, S1A, and S1B). Skipping of exon 9 was observed in ∼60% of the reads. This exon 9 skip would result in an in-frame deletion of 30 amino acids (r.892_984del, p.Ala298_Lys328del) (Figures 1C and 2B). Re-analysis of WES data revealed an intronic homozygous variant in intron 8 of *NDC1*, c.892-21G>A, in both siblings. This variant is absent in gnomAD (v.2.1.1)^24^ and both parents were heterozygous carriers. Splice prediction software predicted that this variant results in a loss of the canonical splice acceptor site (SpliceAI: Δ score = 0.27; Δ score > 0.2 indicates potential effect on splicing, likely by creating an AG dinucleotide in the polypyrimidine tract between the splice acceptor and branchpoint, an alteration able to disrupt the canonical splice acceptor site) (Table S2; Figure S1C).^25^ A minigene assay confirmed that the c.892-21G>A variant indeed caused skipping of exon 9 (Figure 2C). In family 2, WES identified the same homozygous variant as identified in family 1 (NM_018087.4(NDC1):c.892-21G>A, r.892_984del, p.Ala298_Lys328del). Homozygosity for this variant was confirmed by segregation analysis of both parents and the healthy sister, who were all heterozygous.

In families 3 and 4 homozygous missense variants were identified. In family 3, WES identified a homozygous missense variant in *NDC1* (NM_018087.5(NDC1): c.1706C>T, p.Ser569Leu in both affected siblings within a large region of autozygosity (Figure 1C). The CADD (combined annotation-dependent depletion) score^26^ of this variant was 28.7 and the variant was predicted to be probably damaging by PolyPhen2,^24^ benign by MutationTaster, and tolerated by SIFT (Table S2). The variant is absent in gnomAD. In family 4, WES identified a homozygous missense variant in *NDC1*: NM_018087.5(NDC1): c.1720G>A, p.Ala574Thr (Figure 1C). The variant is absent in gnomAD^27^ and its CADD score is 27.1. The pathogenicity prediction program PolyPhen2 reports probably damaging effects, and SIFT classifies the variant as “tolerated” (Table S2).

### Pathogenic *NDC1* variants affect interactions with ALADIN and NPC assembly

**Figure 3:**
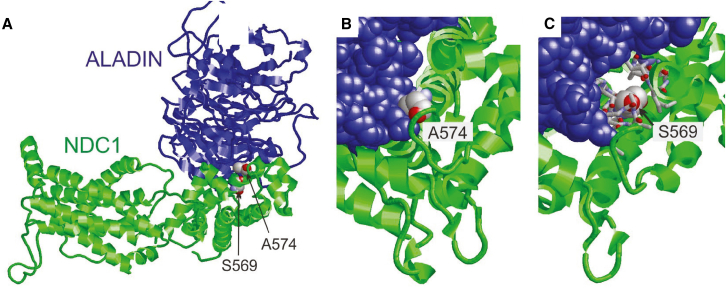
Modeling structural effects of missense variants in NDC1 (A) View of the ALADIN-NDC1 heterodimers of the *Xenopus laevis* NPC. NDC1-ALADIN heterodimer showing that p.Ser569 and p.Ala574 are located close to the dimer interface. This panel is adapted from Huang et al.^28^ (B) Close-up of p.Ala574 showing that this residue forms direct contacts with the ALADIN subunit (shown in blue space-filled presentation). The bulkier substituted threonine side chain is predicted to cause steric clashes with ALADIN thereby decreasing heterodimer stability. (C) Close-up of p.Ser569 showing that this residue forms tight intramolecular interactions (interacting residues shown in stick presentation). The bulkier substituted leucine side chain is anticipated to cause steric clashes thereby decreasing NDC1 stability and consequently its ability to interact with ALADIN. Overall, both variants are located in close proximity to the NDC1-ALADIN interface, suggesting that they have a negative effect on heterodimerization and on the anchoring of the NPC.

To verify the pathogenic effects of the missense variants identified in families 3 and 4, we modeled these variants into the *in silico* NDC1 protein structure. In addition, we modeled the interaction of NDC1 with ALADIN, NUP93, and NUP155 and evaluated the effects of the *NDC1* variants on its interactors (Figure 3A). Interestingly, both missense variants p.Ser569Leu and p.Ala574Thr are located in the C-terminal domain of NDC1 that interacts with ALADIN (Figure S2).^28^^,^^29^^,^^30^ The p.Ala574 residue has been shown to directly interact with ALADIN. Replacement of the alanine residue with threonine is predicted to induce steric clashes with ALADIN, thereby decreasing heterodimer stability (Figure 3B). The p.Ser569 residue does not directly interact with ALADIN, but forms tight intramolecular interactions. Substitution with leucine is predicted to form steric clashes thereby decreasing NDC1 stability and consequently its ability to interact with ALADIN (Figure 3C). Overall, both variants are located in close proximity to the NDC1-ALADIN interface, suggesting that they have a negative effect on heterodimerization and anchoring of ALADIN in the NPC.

**Figure 4:**
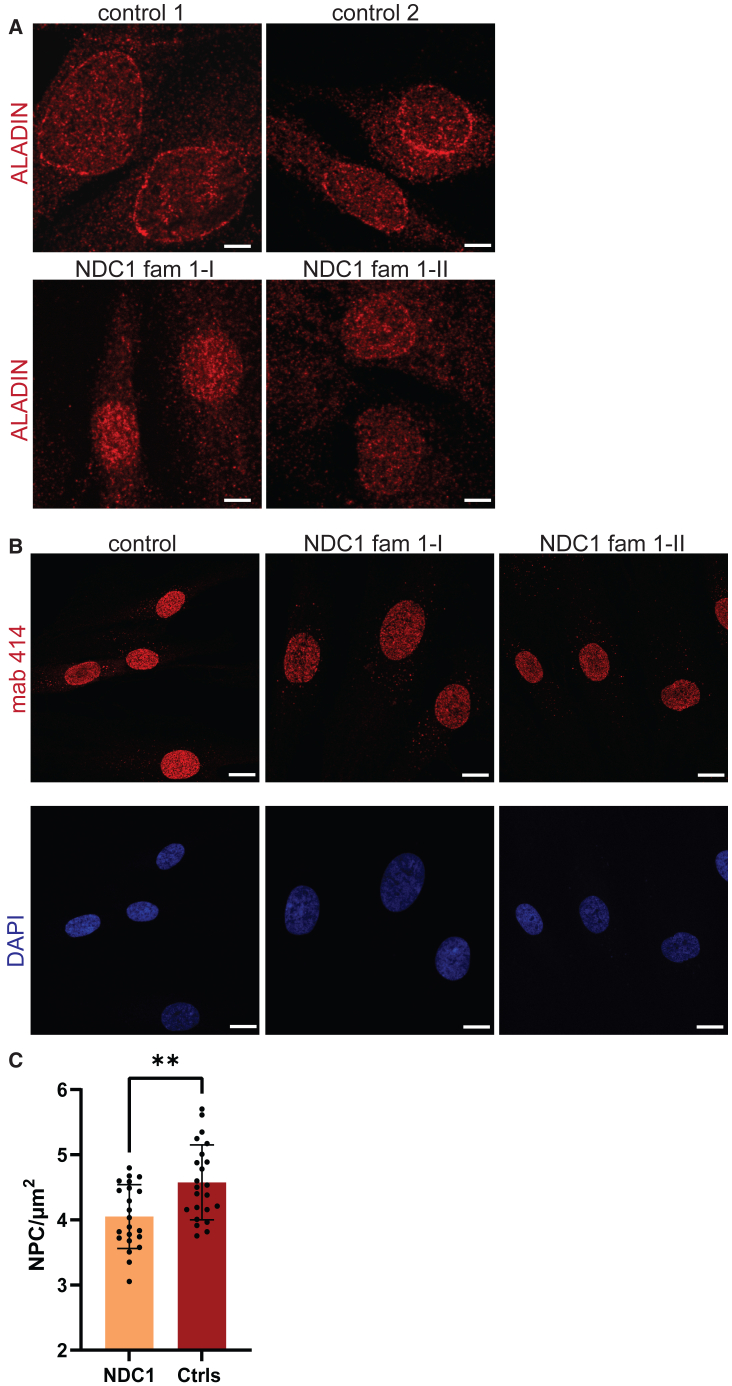
NDC1 variants interfere with ALADIN recruitment and NPC insertion (A) Cell lines were stained with anti-ALADIN antibodies (red) and its localization was evaluated with confocal microscopy. *n* = 2 independent experiments, >10 fields per cell line were evaluated, *n* = 2 *NDC1* individuals; family 1-I and family 1-II. (B) The number of NPCs was examined upon staining with the Mab-414 antibody (red). Nuclei were counterstained with DAPI. The structure of the NE and NPC localization were evaluated with confocal microscopy. (C) Quantification of the number of NPCs in post mitotic cells (CDT1+) with 3D SIM microscopy after staining with the Mab-414 antibody (red). *n* = 3 experiments, *n* = 2 *NDC1* individuals, *n* = 2 controls, two tailed unpaired t test, error bars represent the standard error of the mean (SEM)). ∗∗*p* < 0.01.

To investigate the effects of the p.Ala298_Lys328del variant on recruitment of ALADIN to the NPC, we assessed the localization of endogenous ALADIN in fibroblasts derived from affected individuals harboring this variant (individual 1-I + 1-II) by confocal microscopy and compared this with age-matched control fibroblast lines. Although, localization of ALADIN to the NE was observed in both control cell lines, fibroblasts containing the p.Ala298_Lys328del variant showed a decrease in ALADIN localization to the NE, indicative of recruitment defect (Figure 4A). These results support the pathogenicity of this variant as well as the importance of the NDC1 C terminus for ALADIN recruitment to the NE.

NDC1 is known to be essential for post-mitotic NE formation and the assembly of new NPCs.^13^ To evaluate the effects of our variants on NPC assembly and NE formation we labeled all NPCs with the mAb414 antibody, which recognizes FG-repeat-containing NPCs. The NDC1 p.Ala298_Lys328del variant did not have major effects on NE morphology and NPC distribution during interphase (Figure 4B). The number of NPCs was additionally quantified with 3DSIM high-resolution microscopy. As NPC assembly can occur post-mitotically and during interphase, we used the post–mitotic marker CDT1 to discriminate between these two processes. Comparison of NDC1 p.Ala298_Lys328del cell lines with three unrelated age-matched controls showed an overall decrease of CDT1-positive NPCs in the NDC1 p.Ala298_Lys328del cell lines (Figure 4C, mean NDC1 p.Ala298_Lys328del = 4.05 pore/μm^2^; mean controls = 4.58 pore/μm^2^, *p* < 0.002, two-tailed unpaired t test). These results suggest that the C-terminal NDC1 p.Ala298_Lys328del variant affects both ALADIN recruitment and post-mitotic NPC formation.

## Discussion

This report provides evidence that biallelic *NDC1* variants can cause a neurological phenotype similar to triple A syndrome. In our series, individuals with homozygous *NDC1* variants present with variable alacrima, achalasia, mild developmental delay/intellectual disability, and peripheral (motor) polyneuropathy, but without endocrine anomalies. We show that the variant identified in families 1 and 2 interferes with the physiological function of NDC1 as it decreases the recruitment of ALADIN to the nuclear rim and impairs post-mitotic NPC insertion.

Triple A syndrome is characterized by the triad of esophageal achalasia, alacrima, and adrenal insufficiency and is associated with biallelic *AAAS* variants. The disease phenotype is variable, the complete triad is observed in 70% of affected individuals, and disease onset is often in first decade of life.^15^^,^^31^^,^^32^ Adrenal failure is common and may be life threatening, but individuals without adrenal failure have also been described.^33^^,^^34^ In addition, various neurological symptoms are reported. Upper and lower motor neuron lesions can cause bulbar symptoms, spasticity, intellectual disability, cognitive decline, and cranial neuropathy.^31^^,^^35^ Progressive peripheral neuropathy of motor and sensory nerves, often demyelinating with secondary axonal degeneration, is one of the hallmarks, but its presence has not been systematically investigated in all subjects with *AAAS* variants.^31^^,^^35^^,^^36^ In contrast to the individuals reported in this paper, nearly all triple A syndrome patients present with the pathognomonic sign of marked hyperreflexia without other signs of spasticity. Additional autonomic dysfunction is observed in 33% of affected individuals, and is characterized by postural hypotension, abnormal cardiovascular responses, anisocoria, abnormal pupillary reflexes, impotence, and alacrima. The name “4A-syndrome” has been introduced for the clinical variant with autonomic dysfunction.^37^

Interestingly, the individuals with biallelic *NDC1* variants showed high phenotypic overlap with those having variants in *AAAS* (Table 2). The majority of the examined individuals with *NDC1* variants presented with alacrima and symptoms of achalasia or dysphagia, recurrent vomiting or inability to swallow hard texture food. The formal diagnosis of achalasia was made in two of the individuals (individuals 3-1 + 3-II). Progression of milder dysphagia into achalasia during adolescence should be considered in younger cases, as this is frequently seen in individuals with *AAAS* variants.^38^ Notably, signs of adrenal insufficiency were not observed in any of the individuals with *NDC1* variants, while endocrine disorders are observed in about 80% of individuals with triple A syndrome.^36^^,^^39^ However, this percentage could reflect an ascertainment bias due to the age of onset and initial symptomatology.

**Table 2:** Comparison of the clinical features observed in individuals with *NDC1* and *AAAS* variants

Clinical manifestations	*NDC1*	*AAAS*
Adrenal insufficiency	–	+
Neurological features		
Dysphagia/achalasia	+	+
Clinical signs of polyneuropathy	+	+/−
Hypotonia	+/−	+/−
Hyporeflexia	+	–
Muscular atrophy	+	+/−
Hypertonia	+/−	+
Tongue fasciculations/atrophy	+/−	+/−
Epilepsy	+/−	+/−
Facial weakness	+/−	+/−
Developmental delay	+	+
Dysautonomia		
Alacrima	+	+
Postural hypotension	–	+/−
Abnormal heart responses	+/−	+/−
Sexual dysfunction	–	+/−
Dermatological manifestations		
Hyperpigmentation	–	+/−
Hyperkeratosis	+/−	+/−
Cutis anserine	–	+/−

– indicates never reported, +/− indicates reported in some individuals, + reported >50%.^14^^,^^15^^,^^16^^,^^18^^,^^32^^,^^33^

Compared with individuals reported with *AAAS* variants, individuals with *NDC1* variants presented with prominent neurological symptoms; however, this could be biased given that *AAAS* variant cases are often ascertained based on the endocrinological problems. All *NDC1* individuals had neurological features from early childhood, while the neurological symptoms in *AAAS*-related triple A syndrome usually occur at the end of the first, or the beginning of the second decade of life.^14^^,^^15^^,^^18^ Like in triple A syndrome, the neurological defects observed in *NDC1* cases affect the central, peripheral, and autonomic nervous system. The most commonly observed features were hypotonia, developmental delay/intellectual disability, demyelinating motor neuropathy with secondary axonal injury, alacrima, and tongue fasciculations.^14^^,^^18^^,^^39^ Alacrima was frequent, but no other autonomic disturbances were observed in the NDC1 cohort except for limited heart rate variability in one individual. Peripheral nerve involvement is described in many individuals with triple A syndrome, and is predominantly characterized as a motor axonal neuropathy, which frequently affects the ulnar nerve.^16^^,^^40^^,^^41^

The transmembrane nucleoporin NDC1 anchors various cytoplasmic nucleoporins in the NE with its N-terminal and C-terminal protein tails.^12^^,^^29^ All variants identified in the affected individuals are located in the C-terminal tail of NDC1, which binds ALADIN and NUP155. The identified missense variants (p.Ser569Leu and p.Ala574Thr) are located in a region that interacts with ALADIN, explaining their deleterious effects. The c.892-21G>A variant results in the frame loss of exon 9 (p.Ala298_Lys328del) in about 60% of *NDC1* transcript. This deletion includes a known ALADIN binding site (Figure S2) and results in reduced ALADIN localization to the NE. This is reminiscent of experimental *NDC1* knockdown, which also causes reduced ALADIN NE localization and being consistent with a loss of function mechanism of the human variants.^29^^,^^30^ Similarly, pathogenic missense variants in *AAAS* result in aberrant ALADIN localization. Therefore, decreased recruitment of ALADIN to the NE has been advocated as the main mechanism underlying *AAAS*-related triple A syndrome.^19^^,^^29^^,^^42^

NDC1 also has a well-established role in post-mitotic NPC assembly and NE formation.^43^ Both the N-terminal and C-terminal tails of NDC1 are indispensable for NPC insertion.^13^ Fibroblasts containing the C-terminal p.Ala298_Lys328del variant showed a lower number of post-mitotically inserted NPCs. Interestingly, we did not observe major anomalies in the structure of the NE in these cell lines during interphase, as previously seen upon siNDC1.^43^ These data support the involvement of C-terminal NDC1 in NPC assembly and show that the p.Ala298_Lys328del variant interferes with established NDC1 functions. This variant probably does not induce major structural NE anomalies during interphase since it does not directly affect the NDC1 transmembrane domains. This will enable the integration of NDC1 into the NPC membrane and could as well allow the establishment of physiological protein interactions required for membrane deforming capacities.^13^

The consequences of impaired ALADIN recruitment to the NPC on cellular homeostasis are unknown. Several studies indicate that NDC1-mediated anchoring of ALADIN at the NPC is essential for selective nuclear import of DNA repair proteins (ligase 1; APRATAXIN) and ferritin heavy chain 1, a protein that protects against nuclear oxidative stress.^44^^,^^45^ Consequently, ALADIN-depleted cells have an altered response to oxidative stress and increased levels of reactive oxygen species.^20^^,^^44^^,^^45^ Moreover, roles for ALADIN unrelated to the NPC have been described. ALADIN assists in mitotic spindle formation by altering the localization of Aurora A,^46^ and it interacts with progesterone receptor membrane component 2, which regulates the activity of cytochrome P450.^47^ Moreover, a recent report focusing on adrenal glands showed a role for ALADIN in the nucleocytoplasmatic transport of cyclic AMP-dependent protein kinase and subsequent dysregulation of the steroidogenic pathway.^48^ The overlap in phenotypes caused by *NDC1* and *AAAS* variants suggests that impaired recruitment of ALADIN to the NPC contributes to the clinical features shared among the two groups of affected individuals, e.g., alacrima, achalasia, and neurological defects. Possibly, functions of ALADIN unrelated to the NPC could be contributing to distinguishing features such as adrenal insufficiency.

Dysregulation of oxidative stress is one of the mechanisms that could contribute to the neurological features observed. While the pathophysiological mechanisms underlying the clinical features observed in triple A syndrome are not completely understood, oxidative stress is often causally related to peripheral neuropathy, and is implicated in several neurodegenerative and neurodevelopmental disorders.^49^^,^^50^ Previous studies have suggested that the triple A-related peripheral neuropathy might result from a defect of ACTH receptors on neurons or glia with secondary demyelination.^17^^,^^36^ However, our results, as well as several *AAAS* case descriptions, do not support this hypothesis as the subjects here reported did show demyelinating neuropathy without endocrine anomalies.

Although about 80% of individuals with triple A syndrome have an *AAAS* mutation, evidence for genetic heterogeneity has been reported for over two decades.^35^^,^^38^ A syndrome of alacrima, achalasia, and mental retardation (OMIM: 615510) has been linked to biallelic variants in the guanosine diphosphate-mannose pyrophosphorylase A (*GMPPA*) gene.^51^ The GMPPA protein inhibits guanosine diphosphate-mannose pyrophosphorylase B, which results in increased GDP-mannose levels and imbalanced glycosylation reactions.^52^ Moreover, variants in *TRAPPC11* cause cerebral atrophy, intellectual disability and movement disorders, scoliosis, achalasia, and alacrima. The *TRAPPC11* variants were also reported to influence protein glycosylation.^53^ Similar to individuals with pathogenic *NDC1* variants, the distinguishing feature of the disorder related to these variants is the absence of adrenocortical dysfunction. Interestingly, the consequences of the variants in *GMPPA* and *TRAPPC11* seem to be unrelated to the NPC structure, which suggests that the molecular mechanism causing the features observed in triple A-related disease are not only directly related to the NPC, but also to secondary defects, for example, alterations in selective nuclear trafficking.

Our observation shows that *NDC1* variants should be considered in individuals with alacrima, achalasia, and neurological defects, mostly severe peripheral neuropathy. The variants identified here in the affected individuals all seem to have an effect on the interaction with ALADIN, explaining the high phenotypic overlap with *AAAS*-related triple A syndrome. The future description and evaluation of additional individuals with *NDC1* variants is essential to better define the associated phenotype and gain more insight into the underlying disease mechanisms.

### Statistics

Statistical analysis was performed using GraphPad Prism v.9.0. All datasets were checked for normality and outliers. All error bars represent the standard error of the mean. Details about the statistical tests are available in the figure legends.

### Data and code availability

The WES and RNA sequencing data supporting the current study have not been deposited in a public repository out of respect for the patients' privacy, but data are available from the corresponding author on request.

## Web resources

OMIM: https://www.omim.org/
Genome Analysis Toolkit (GATK): http://www.broadinstitute.org/gatk/
Burrows-Wheeler Aligner: http://bio-bwa.sourceforge.net/
AlphaFold: https://alphafold.ebi.ac.uk/
GeneMatcher: https://genematcher.org/statistics/
GnomAD: https://gnomad.broadinstitute.org/
Web browser application for RNA-seq analysis: https://github.com/KlinGenErasmusMC/rnaseq-voila

## Supplemental information

Data S3. Table S3

Document S2. Article plus supplemental information
